# FAIRY: a randomized controlled patient-blind phase III study to compare the efficacy and safety of intravenous ferric carboxymaltose (Ferinject®) to placebo in patients with acute isovolemic anemia after gastrectomy - study protocol for a randomized controlled trial

**DOI:** 10.1186/1745-6215-15-111

**Published:** 2014-04-05

**Authors:** Daniel Reim, Young-Woo Kim, Byung Ho Nam, Mi-Jung Kim, Jeong Hwan Yook, Young Kyu Park, Sung Hoon Roh, Wan Sik Yu, Jae Moon Bae

**Affiliations:** 1 Gastric Cancer Branch, Research Institute & Hospital, National Cancer Center, 323 Ilsan-Ro, Ilsandong-Gu, Goyang-si, Gyeonggi-do 411-769, Republic of Korea; 2 Department of Surgery, Klinikum Rechts der Isar der Technischen Universität München, Ismaninger Strasse 22, 81675 Munich, Germany; 3 National Cancer Center, Biometric Research Branch, Research Institute for National Cancer Control & Evaluation, 323 Ilsan-Ro, Ilsandong-gu, Goyang-si, Gyeonggi-do 411-764, Republic of Korea; 4 Department of Surgery, Samsung Medical Center, Sungkyunkwan University School of Medicine, 81 Irwon-Ro, Gangnam-gu, Seoul 135-710, Republic of Korea; 5 Asan Medical Center, 88 Olympic-Ro 43-Gil, Songpa-gu, Seoul 138-736, Republic of Korea; 6 Chonnam National University Hwasun Hospital, 322 Seoyang-Ro Hwasun-Eup, Hwasun-Gun, Jeonnam 519-763, Republic of Korea; 7 Yonsei University Health System Severance Hospital, 50 Yonsei-Ro, Seodaemun-gu, Seoul 120-752, Republic of Korea; 8 Gastric Cancer Center, Kyungpook National University Medical Center, 807 Hogukno, Buk-gu, Daegu 702-210, Republic of Korea

**Keywords:** Ferric carboxymaltose, Isovolemic postoperative anemia, Gastric cancer, Randomized controlled trial

## Abstract

**Background:**

Isovolemic anemia (decrease in hemoglobin concentration with normal or even increased blood volume) after gastric cancer surgery may negatively influence short- and long-term outcomes. Therefore correction of isovolemic postoperative anemia is supposed to be beneficial. This prospective randomized placebo-controlled multicenter trial is designed to evaluate the efficacy of ferric carboxymaltose administration with the primary end point of successful hemoglobin level increase by 2 g/dl at 12 weeks after randomization.

**Methods and design:**

Gastric cancer patients after oncologic resection and postoperative hemoglobin level ≥ 7 g/dl to <10 g/dl at postoperative days 5 to 7 will be eligible for trial inclusion. After randomization, 450 patients (225 per group) are going to be subjected either to administration of ferric carboxymaltose (treatment group) or normal (0.9%) saline (placebo group). Patients will be blinded to the intervention. Patients will undergo evaluation for hemoglobin level, hematology and quality of life assessment 3 and 12 weeks after randomization.

**Discussion:**

Correction of isovolemic postoperative anemia in gastric cancer patients after oncologic resection is considered to be beneficial. Administration of ferric carboxymaltose is considered to be superior to placebo for anemia correction without the possible risks of red blood cell transfusion. Further, improved quality of life for patients with quick recovery of hemoglobin levels is expected.

**Trial registration:**

NCT01725789 (international: http://www.clinicaltrials.gov) and NCCCTS-12-644 (NCC, Korea).

## Background

Perioperative anemia occurs in 25% to 75% of cancer patients, and the prevalence of anemia in the immediate postoperative period after major surgery is as high as 90% [1,2]. Postoperative acute isovolemic anemia (decrease in hemoglobin concentration with normal or even increased blood volume) can affect the recovery and quality of life (QoL) of patients by subtly slowing the reaction time, deteriorating memory, increasing heart rate, and decreasing oxygen levels [3,4]. It has been suggested that low hemoglobin levels may affect oncologic outcomes in gastric cancer patients [5,6]. Jung et al. revealed that low hemoglobin levels were related to postoperative complications [5]. Further, it was demonstrated that postoperative anemia resulted in decreased overall survival [6]. Therefore correction of postoperative anemia is an important issue. However, transfusion guidelines and patient blood management programs are not commonly implemented so far. This is reflected by the fact that postoperative transfusion rates for colorectal and gastric cancer patients were reported to be 10% to 38% [7,8]. It was demonstrated before that transfusion of red blood cells (RBC) carries substantial risks for patients and may even worsen postoperative oncologic longterm outcome [9,10]. Those risks may be acute hemolytic reactions, transfusion-related acute lung injury, immunization events, organ dysfunctions, and failures and the possibility of incorrect transfusions. The best evidence so far is derived from a meta-analysis for red blood cell transfusions in colorectal cancer patients which concludes that RBC transfusion is related to higher cancer recurrence and decreased survival rates [11]. This data was also confirmed for other tumor entities such as pancreatic cancer and hepatocellular carcinoma [12,13]. Therefore alternative procedures to classical RBC-transfusion should be considered of highest interest. Iron supplementation may be a conceivable alternative. However, oral iron intake was reported to be related to a considerable amount of side effects [14] which can be hypersensitivity, paresthesia, hypotension, dyspepsia, vomiting, abdominal pain, myalgia/arthralgia, and pyrexia. Further, dietarian supplementation may be hampered by substantially affected resorption from the GI-tract after gastric cancer surgery [14]. Therefore intravenous iron supplementation is a substantial alternative. However, iron dextran infusion is considered to be rarely related to potentially life-threatening side effects [15]. Alternatively ferric carboxymaltose was proposed to be safer regarding the common side effects of intravenous (iv-) iron infusion [15].

In a retrospective observational study performed at the sponsor site the role of intravenous iron was assessed before [16]. Postgastrectomy patients treated with iron sucrose had a faster and more complete Hb-response compared to untreated patients and none of the treated patients developed chronic anemia 12 months after surgery. Further, no adverse events related to iron infusion were noted. However, selection bias could not be ruled out in this study, because in this retrospective analysis the percentage of advanced gastric cancer patients treated by intravenous iron was higher than in the non-treated group. Therefore we propose this randomized placebo controlled phase III trial in order to evaluate the efficacy and safety of intravenous ferric carboxymaltose supplementation for correction of postoperative isovolemic anemia in gastric cancer patients.

## Methods

### Study population, inclusion, and exclusion criteria

**Figure 1:**
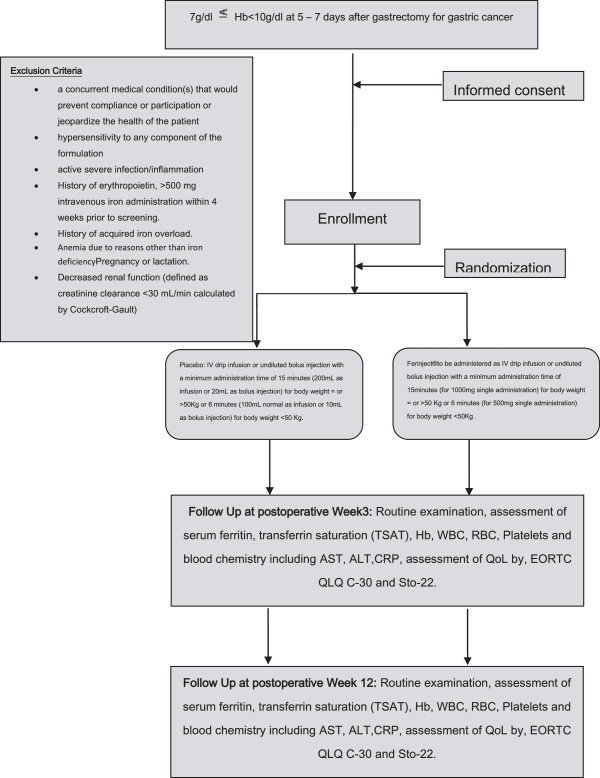
Study scheme of FAIRY trial, inclusion/exclusion criteria, intervention, and end points.

All patients with a hemoglobin-level ≥ 7 g/dl to <10 g/dl at postoperative days 5 to 7 after oncologic gastrectomy for gastric cancer will be eligible for screening. All patients aged 20 years and over and willing to give informed consent will be eligible. Participation in another trial interfering with the outcome of this study, language problems, lack of compliance, mental inability, unstable angina or myocardial infarction within 6 months of the trial, severe respiratory disease, ASA (American Society of Anesthesiologists) Score >3, inadequate liver, kidney, and bone-marrow functions (evaluated by measurement of white blood cell count (WBC), platelets, aspartate aminotransferase (AST), alanine aminotransferase (ALT), C-reactive protein (CRP), and creatinine), pregnancy/lactation, and Eastern Cooperative Oncology Group (ECOG) status >1 will be considered unspecific exclusion criteria. Further, patients with a known history of hypersensitivity or allergy to ferric carboxymaltose, active inflammatory disease (surgical, site infection, pneumonia, Systemic Inflammatory Response Syndrome (SIRS)), history of previous erythropoietin or iv-iron administration within 4 weeks prior to screening, history of iron overload (hemochromatosis) and anemia due to reasons other than iron deficiency will not be eligible for this trial. The study protocol was approved by the Institutional Review Board (IRB) of the National Cancer Center Korea on 7 November 2012 (NCCCTS-12-644). All participating centers approved the trial by their local IRBs subsequently. Further, the trial was registered in the clinical trials database (NCT01725789) (Figure 1).

### Procedures

#### General procedures

After consenting to participate in the trial, patients will be randomized to either one of the two treatment groups (500 to 1,000 mg ferric carboxymaltose (Ferinject®) *vs.* placebo (0.9% saline)). After inclusion of eligible patients medical history will be taken and all concomitant diseases and medications will be recorded. All trial participants will receive routine examination and blood testing for the following parameters: hemoglobin, RBC, WBC, platelets, serum ferritin, transferrin saturation, AST, ALT, CRP, and creatinine.

#### Assessment of quality of life

Quality of life (QoL) as one of the most important secondary endpoints will be assessed by standardized European Organization for Research and Treatment of Cancer (EORTC) QLQ C-30, and STO-22 (EORTC Study Group on Quality of Life) questionnaires, which are already broadly established and validated. Crawford and Cella [17] reported that the maximal incremental gain in QoL occurs when the hemoglobin level lies in the range of 11 to 13 g/dL. Differences in QoL were even noted when the Hb-level was only 1 g/dL lower. Therefore we hypothesize that rapid Hb-nomalization by intravenous iron administration may improve QoL. As such, QoL will be assessed at multiple time points postsurgery during the follow-up visits.

#### Intervention

The treatment group will be administered ferric carboxymaltose (1,000 mg for bodyweight ≥50 kg or 500 mg for bodyweight <50 kg (maximum dose of ferric carboxymaltose: 20 mg/kg)) as iv-drip infusion over an administration time of at least 15 (1,000 mg) or 6 (500 mg) min. All patients with a serum ferritin level of <15 ng/mL and Hb-level <10 g/dl at postoperative week 3 will receive a second administration of 500 mg ferric carboxymaltose as described above. The placebo group will receive normal saline (0.9%) as iv-drip infusion (200 mL for bodyweight ≥50 kg or 100 mL for bodyweight <50 kg) over an administration time of at least 15 (200 mL) or 6 (100 mL) min. During infusion patients will be blinded.

#### Follow-up

Follow-up visits will take place at 3 and 12 weeks (±7 days) after the study intervention. On the follow-up visits patients will undergo blood testing and QoL evaluation as described above.

### Study objectives

#### Primary endpoint

The primary efficacy endpoint will be the number of responders defined as an increase of the hemoglobin level of at least 2 g/dl related to the baseline hemoglobin measured after randomization at 12 weeks after administration of either placebo or ferric carboxymaltose.

#### Secondary endpoints

Major secondary endpoints will be: percentage of patients with Hb ≥10, 11, and 12 g/dL at 3 and 12 weeks (independent of alternative anemia management including transfusion or use of erythropoietin stimulating agents), percentage of patients requiring alternative anemia management therapy, average time-to-response (Hb increase ≥2 g/dL and/or Hb ≥10 g/dL) (independent of alternative anemia management including transfusion or use of erythropoietin stimulating agent, development of Hb, ferritin, and transferrin saturation over the study duration (12 weeks) independent of alternative anemia management including transfusion or use of erythropoietin stimulating agents and finally safety/tolerability of ferric carboxymaltose. QoL as one of the most important secondary endpoints will be assessed by EORTC QLQ C-30 and STO-22 questionnaires at 3 and 12 weeks.

### Methods against bias

Randomization into two treatment groups will be performed in order to omit selection bias from this trial. Randomization will be performed as block randomization in fixed block sizes in a 1:1 allocation ratio using a centralized web-based randomization system (eVelos (http://eresearch.ncc.re.kr/eres/jsp/ereslogin.jsp)). In order to achieve equal group sizes randomization will be stratified for each respective center (six centers). Further, patients will be stratified according to the need of adjuvant chemotherapy depending on the surgical/pathological stage (adjuvant CTx in patients with clinical/surgical/pathological stages II to IV according to UICC/AJCC [18]). Patient blinding will rule out possible influences on QoL assessment. Patients and observers for postoperative outcomes will be blinded to guarantee an optimal study outcome. Potential confounding will be reduced by the randomization process.

### Sample size calculation, statistical considerations, and trial feasibility

The sample size is based on a superiority design assuming a response rate to ferric carboxymaltose of 75% (per primary endpoint definition) by week 12. A response rate of 60% is assumed in the control group. This results in a 15% advantage for the treatment group which is considered medically meaningful. In order to achieve a statistically significant result with 90% power at a significance level of α = 0.05, 400 patients are required to be randomized according to the Pocock formula [19]:

$$n=f\left(\alpha ,\beta \right)\times \left[p1\times \left(100-p1\right)+p2\times \left(100-p2\right)\right]/{\left(p2-p1\right)}^{2}$$

where p1 and p2 are the response rates in the control and experimental groups, respectively, and f(α, β) = [*Φ*^- 1^(α/2) + *Φ*^- 1^(β)]^2^.

To account for potential patient drop-outs over the 12-week study period, the sample size is estimated at 450 patients (225 per group). Although this number appears to be rather high, recruitment should be accomplished within several months. Each of the six participating centers treats at least 500 patients for gastric cancer every year which sums up to a volume of 3,000 patients per year. Based on the assumption of an incidence rate of 15% for postoperative anemia (18% in the sponsor’s institution) 400 patients may be recruited within one year.

The outcome parameters will be analyzed by a Pearson chi-square test or Fisher’s exact test (patient age and gender, clinicopathologic data, and morbidity) and Student’s t-test (Hb-level before treatment and hospital days after treatment). The Z-test will be used to determine whether or not a significant difference exists between two groups during follow-up.

### Safety

Stopping rules of the study medication will not apply due to a single administration of ferric carboxymaltose. However, in case of hypersensitivity or anaphylactic reactions immediate interruption of infusion will be mandatory. Further management will be left to the discretion of the physician on duty. Any (serious) adverse event ((S) AE), any (unexpected) (serious) adverse drug reaction, and any suspected unexpected serious adverse reaction (SUSAR) will be documented. All SAEs, SADs, and SUSARs will have to be documented and reported to the IRB by the responsible investigator according to local regulations.

### Documentation and data handling

All information required by the study protocol and collected during this trial will be entered in the electronic case report form (eCRF) using the web-based eVelos system (http://eresearch.ncc.re.kr/eres/jsp/ereslogin.jsp). The completed eCRFs will be reviewed and signed by the investigator or subinvestigator and sent to the independent data management group in the eVelos team. Quality control is going to be enforced by site visits and CRF review. The data will be handled, managed, and analyzed according to appropriate regional standard operating procedures. The study will be conducted in accordance with ICH-GCP and the Declaration of Helsinki.

## Discussion

Postoperative anemia occurs in around 18% of patients after oncologic gastrectomy [16]. This signifies a considerable amount of patients in need of hemoglobin correction and prevention of chronic anemia. For gastric cancer patients high level evidence derived from prospective studies on anemia correction other than RBC transfusion are not available yet. Therefore we propose this prospective randomized placebo controlled trial.

Primary efficacy endpoint of this prospective study is the number of treatment responders after administration of either placebo or ferric carboxymaltose. Response is defined as an increase of the hemoglobin level of at least 2 g/dl related to the baseline hemoglobin 12 weeks after randomization. A hemoglobin level of 10 g/dl will be used as a cutoff value for our study based on the guidelines published by the American Society of Clinical Oncology and the American Society of Hematology for the treatment of cancer-related anemia, recommending Hb <10 g/dl as a treatment threshold [20]. The increase of 2 g/dl was chosen on the basis of the former FERGIcor trial [21], which defined treatment responders to ferric carboxymaltose when the hemoglobin level increased by 2 g/dl 12 weeks after initiation of treatment and revealed significant improvements in QoL.

After gastrectomy, damage on the gut may impact the ability of the patient to absorb iron from diet, limiting correction of iron deficiency anemia [22]. Therefore an intravenous compound was chosen for this trial. To date, over 3,600 subjects were treated by ferric carboxymaltose in various clinical studies. Across those clinical studies [14,15,21,23], replenishment of iron stores was consistently observed. In subjects with more severe or prolonged iron deficiency, anemia correction using ferric corboxymaltose consistently resulted in medically significant increases of Hb values. This improvement was usually found within 2 weeks. In addition to the correction of laboratory parameters, iron replacement therapy demonstrated significant improvements of QoL and functional status. In phase III studies, ferric carboxymaltose (Ferinject®) was reported to have improved tolerability and fewer side effects than iron sucrose [24] together with faster correction of postoperative anemia.

Postoperative anemia may increase tumor resistance to possibly necessary adjuvant chemotherapy in advanced gastric cancer patients. It was found that anemia is the strongest prognostic factor for oncologic outcomes of 5-fluorouracil-based chemotherapy [25]. Anemic patients revealed significantly lower response rates and overall survival rates than those with regular Hb-levels. Therefore it is of utmost interest to improve Hb-levels for postoperative AGC patients who are supposed to undergo adjuvant chemotherapy in a curative setting. Besides that, baseline Hb may be lower for advanced gastric cancer patients due to a higher risk of blood loss due to more invasive surgery (open procedure, more aggressive lymph node dissection, and longer operating time) compared to early gastric cancer patients. As the improvements on QoL may be more pronounced in this group, AGC patients will have to be stratified in a subgroup analysis.

Conclusively there is emerging evidence that patient blood management for postoperative isovolemic anemia may be superior to just observation or red blood cell transfusion. The proposed study represents a well powered randomized placebo-controlled trial in order to elucidate the influence of ferric carboxymaltose substitution on correction of isovolemic anemia and QoL in gastric cancer patients.

## Trial status

Recruiting. The first patient was randomized in January 2013.

## Abbreviations

AE: Adverse event; AGC: Advanced gastric cancer; ALT: Alanine aminotransferase; AST: Aspartate aminotransferase; CRP: C-reactive protein; EGC: Early gastric cancer; ESA: Erythropoiesis stimulating agent; FCM: Ferric carboxymaltose; FU: Fluorouracil; GGT: Gamma-glutamyl transpeptidase; Hb: Hemoglobin; HCG: Human chorionic gonadotropin; ID: Iron deficiency; IDA: Iron deficiency anemia; LDH: Lactate dehydrogenase; OS: Overall survival; PFS: Progression-free survival; QoL: Quality of life; SAE: Serious adverse event; SUSAR: Suspected unexpected serious adverse reaction; TSAT: Tranferrin saturation; TIBC: Total iron binding capacity.

## Competing interests

The authors declare that they have no competing interests.

## Authors’ contributions

DR was responsible for drafting the manuscript and critical revision, data collection, and interpretation and final approval of the manuscript. YWK was the principal investigator responsible for conception and design, data collection and analysis, critical revision, and final approval of the manuscript. BHN was the trial statistician, responsible for conception and design, critical revision, data collection and analysis, and final approval of the manuscript. MJK was responsible for conception and design, critical revision, data collection and analysis, and final approval of the manuscript. JHY, YKP, SHR, WSY, and JMB were responsible for critical revision, data collection and analysis, and final approval of the manuscript. All authors read and approved the final manuscript.
